# Traceability of Rizhao green tea origin based on multispectral data fusion strategy and chemometrics

**DOI:** 10.1016/j.fochx.2025.102346

**Published:** 2025-03-18

**Authors:** Mengqi Guo, Zhiwei Chen, Zezhong Ding, Dewen Wang, Dandan Qi, Min Lu, Mei Wang, Chunwang Dong

**Affiliations:** Tea Research Institute, Academy of Agricultural Machinery Sciences, Shandong Academy of Agricultural Sciences, Jinan 250100, China

**Keywords:** Rizhao green tea, Origin traceability, Multispectral, Data fusion, Chemometrics

## Abstract

This study proposes a novel method that combines multispectral data fusion strategies with chemometric analysis for the origin traceability of Rizhao green tea. The research found significant differences in the sensory scores and key physicochemical components (catechins, caffeine, and amino acid content) between Rizhao green tea and tea from southern China. By integrating data from near-infrared and hyperspectral technologies, the prediction accuracy of multivariate models (including Partial Least Squares Regression (PLSR), Support Vector Regression (SVR), Random Forest (RF), and Convolutional Neural Networks (CNN)) was improved. The performance of the fused dataset outperformed single-spectral datasets. The study found significant spectral differences in tea samples from different regions, leading to robust differentiation. Both SVM and RF discriminant models based on near-infrared spectral data achieved 100 % accuracy. This method provides a reliable and efficient tool for green tea traceability, with potential applications in quality control and authenticity verification within the tea industry.

## Highlights

• Revealed sensory-physicochemical component correlations in Rizhao green tea.
• Rizhao green tea's key components surpass southern China's low-latitude teas.
• NIR outperforms VIS in Rizhao tea origin discrimination accuracy.
• Multi-spectral data fusion enhances predictive model performance.

## 1 Introduction

Tea is one of the three major beverages consumed worldwide, the other two being coffee and cocoa. Rizhao, located at 35 degrees north latitude on the coast of the Yellow Sea, has a warm temperate humid–monsoon climate. It has an abundance of sunlight, plentiful rainfall, and mountainous and hilly soils that are mildly acidic. The exceptional climatic conditions and geographical location give Rizhao green tea its unique qualities of “thick leaves, strong flavor, enhanced aroma, and resistance to brewing”. Thus, Rizhao green tea is regarded as the “premier tea in the northern region of the Yangtze River” (Ao Sun et al., 2023). Currently, the market conditions for Rizhao green tea are unfavorable, with inconsistent quality, counterfeit and substandard tea products, and erratic pricing. As a result, Rizhao green tea has attained a state of stagnation in the tea industry. The appearance and production process of green teas from different origins are similar, making it difficult for consumers to distinguish Rizhao green tea by sensory means such as appearance, aroma, and flavor.

The flavor of tea is influenced by the concentrations of amino acids, catechins, alkaloids, and crude fiber, which are constituents of tea's rich intrinsic chemical composition. Amino acids are the main contributors to the refreshing flavor of tea. While tea polyphenols are a collective term for over 30 polyphenolic compounds, mainly catechins, which are beneficial to the human body, such their antioxidant qualities, lowering blood pressure, and lipid-lowering effects (He et al., 2022; Orita et al., 2023). Alkaloids are important compounds in tea, and together with tea polyphenols constitute the main active components in tea. Among them, Caffeine in purine bases is one of the most representative alkaloids, which has a refreshing and diuretic effect (Yong et al., 2022). Crude fiber cannot be digested and absorbed in the human body, but it can promote gastrointestinal motility and aid digestion when consumed in moderation (Śmiechowska & Dmowski, 2006). Green teas from different regions have distinct chemical compositions due to factors such as climate conditions, altitude, and latitude. Therefore, the origin of Rizhao green tea can be traced based on the content of its major chemical compounds. These chemical compounds directly affect the aroma, taste, and appearance of tea, which are key factors in sensory evaluation. The primary method used to evaluate the quality of traditional tea is manual sensory evaluation, in which qualified national tea tasters use their senses to analyze the origin and quality of tea. Green tea's chemical composition must be measured precisely using tools like UV spectrophotometers, which cannot detect changes in a matter of seconds. Therefore, establishing a nondestructive, rapid, inexpensive, and environmentally friendly identification method for Rizhao green tea plays an extremely important role in combating counterfeit and substandard Rizhao green tea in the market.

Chemometrics and spectroscopy together can be a valuable tool for determining the provenance of tea (Pan, Liu, & Chi, 2023). Near-infrared spectroscopy (NIRs) technology is characterized by its rapid and non-destructive nature. Its spectra primarily reflect the vibrational absorption of internal atoms such as carbon, hydrogen, oxygen, and nitrogen, carrying rich chemical information. It has applications in agriculture and food, healthcare, environmental monitoring, industry, and chemical engineering (Liu et al., 2023; Ouyang, Fan, Chang, Shoaib, & Chen, 2025). The advantages of spectrum technology and image information are combined in hyperspectral imaging (Chen et al., 2024; Sun et al., 2021; Wang et al., 2024) technology. It possesses the benefits of efficiency, objectivity, accuracy, and nondestructiveness. It is utilized in the evaluation of the tea's internal quality components, variety detection, year identification, grade classification, and origin identification (Li et al., 2021; Ye Seong Kang et al., 2022; Amit Kumar et al., 2013). For the purpose of identifying green tea, Ying Liu and colleagues (Liu et al., 2022) Investigated the viability of combining chemometrics and visible-infrared hyperspectral imaging (NIR-HSI). To reduce the dimensionality of the spectra of tea samples from three different regions in Chongqing, they used principal component analysis (PCA). Using nonlinear SVM algorithms and linear partial least-squares discriminant analysis (PLS-DA), they created discriminant models. The highest accuracy of the prediction set was 97.5 %. Puneet Mishra et al. (Puneet Mishra, Nordon, Asaari, Lian, & Redfern, 2019) utilized 16 green tea products sourced from seven different countries. They sharpened and enhanced the texture details of the images and then utilized a moving window operation to extract texture information from the statistical properties of the gray-level cooccurrence matrix of the sharpened image plane. To improve classification performance, spectral data and texture information were fused as a preprocessing step and then used as input for the support vector machine (SVM) model. The results indicated that the fusion modeling of spectral and texture information can improve the classification of 16 green tea products when compared to models established using only spectral or texture information. However, research on the fusion of multispectral and color texture features combined with chemometric analysis for tracing the origin of Rizhao green tea remains limited.

To address the challenges in accurately tracing the geographical origin of Rizhao green tea, this study utilizes a multispectral data fusion strategy (VNHI) combined with chemometric methods. This approach aims to overcome the complexity of existing origin traceability techniques. Near-infrared (900–1700 nm, NI) and hyperspectral (300–1000 nm, VI) data of tea samples are collected, and the color and texture features (TEX) of the tea samples are extracted. Principal Component Analysis (PCA) combined with six different data fusion techniques (NI, VI, NI + VI, NI + TEX, VI + TEX, NI + VI + TEX) is used to create origin identification models. SVR prediction models for the sensory scores and key chemical compounds of Rizhao green tea are established using four different preprocessing methods combined with six data fusion strategies to optimize the best combination. Finally, linear and nonlinear prediction models are developed to identify the optimal predictive model. This approach provides a reliable method to trace the origin of Rizhao green tea.

## 2 Materials and methods

### 2.1 Tea sample preparation

A total of 105 tea samples were analyzed in this study. Among them, 60 samples were sourced from Shandong Province (SD), including 25 from Linyi City (LY, 35°N, 118°E), 15 from Qingdao City (QD, 36°N, 120°E), and 20 from Rizhao City (RZ, 35°N, 119°E). Additionally, 15 samples each were collected from Yibin City (SC, 28°N, 104°E) in Sichuan Province, Lishui City (ZJ, 28°N, 119°E) in Zhejiang Province, and Zunyi City (GZ, 27°N, 107°E) in Guizhou Province—three of China's major tea-producing regions. All tea samples were processed in local tea factories following the standard production protocol for Rizhao green tea, which includes withering, pan-firing, rolling, and drying. The final products were all rolled green tea. To ensure sample integrity, all tea samples were vacuum-sealed, stored, and transported at −5 °C until analysis.

### 2.2 Sensory evaluation method

According to the requirements of GB/T 23776–2018 Sensory Evaluation Methods of Tea, the Tea Research Institute of the Shandong Academy of Agricultural Sciences conducted a sensory evaluation of 105 tea samples. The evaluation was performed by three certified senior tea reviewers from the Green Tea Sensory Review team. To assess the dried tea's appearance, a 100 g sample was placed on an evaluation tray. For brewing, 3 g of tea was steeped in 150 mL of boiling water for 4 min. Panelists sequentially evaluated the brewed tea's infusion color, aroma, flavor, and infused leaf, assigning scores to each sensory attribute. The total sensory score was then calculated based on the individual scores for appearance, infusion color, aroma, flavor, and infused leaf, using the following formula:

(1) $$S\_T=\sum_{i=1}^{5}z_{i}\times s_{i}$$

where the weights (Si = {flavor, appearance, aroma, infusion color, infused leaf}) for each of the five sensory scores are Zi = {0.30, 0.25, 0.25, 0.10, 0.10}. S_T represents Total Score, which refers to the overall score from the sensory evaluation of tea.

### 2.3 Determination of physicochemical components in tea

The contents of catechins and caffeine were determined in accordance with GB/T 8313–2018 (Determination of Total Polyphenols and Catechins in Tea) and GB/T 8312–2013 (Determination of Caffeine Content in Tea). High-performance liquid chromatography (HPLC) was employed to analyze caffeine and catechins concentrations. The separation was performed using a C18 liquid chromatography column (particle size: 5 μm, 250 mm × 4.6 mm) maintained at 35 °C. The mobile phase consisted of 2 % acetic acid (Phase A) and 100 % acetonitrile (Phase B), with a flow rate of 1.0 mL/min. The detector wavelength was set at 268 nm, and the injection volume was 10 μL. The elution process was conducted as follows: from 0 to 16 min, the proportion of Phase B increased from 6.5 % to 15 %; from 16 to 25 min, it further increased to 25 %; between 25 and 25.5 min, it gradually decreased back to 6.5 % and remained at this level from 25.5 to 30.0 min. Quantification was performed using the external standard method. Additionally, the total free amino acid content and crude fiber content were determined following GB/T 8314–2013 (Determination of Free Amino Acid Content in Tea) and GB/T 8310–2013 (Determination of Crude Fiber Content in Tea), respectively.

### 2.4 Collection of near-infrared spectroscopy and hyperspectral imaging systems

#### 2.4.1 Collection of NIR data

The near-infrared (NIR) spectra of tea samples were collected using the IAS3100 NIR spectrometer from Wuxi Intelligent Analytical Service Co., Ltd., China. Diffuse reflectance spectral scans of Rizhao green tea and other tea samples were conducted at room temperature. The laboratory relative humidity was maintained at around 60 %, and the temperature was kept around 20–25 °C. To ensure the stability of the spectrometer during data collection, the tea samples in the petri dishes were compacted to ensure uniformity. During the spectral scanning process, tea samples are placed in a round petri dish to be fully compacted. Turn on the instrument 30 min before starting data collection to allow the device to reach a “stable state”. Each tea sample was scanned 10 times, and the average of these 10 scans was used as the final spectral data for that sample. A total of 105 spectral datasets were collected to establish a classification prediction model. The spectral range detected was from 900 to 1700 nm, with a total of 801 spectral points.

#### 2.4.2 Hyperspectral image acquisition

ISpecHyper-VS1000-Lab from Lyson Optics (Shenzhen, China) was utilized for hyperspectral imaging. The equipment included a computer, an integrated hyperspectral dark box (1200 × 500 × 500 mm), a hyperspectral camera, a light source, and a linear displacement sampling platform. The halogen light source was positioned 30 cm above the sample scanning platform, with the light directed downward. The hyperspectral camera and the halogen light source were on the same horizontal plane. The spectral resolution was 2.5 nm, and the spectral range detected was 300–1000 nm, comprising 300 wavelength points. After that, petri dishes were set up on the platform within a dark box to capture hyperspectral images. Each tea sample (20 g) is placed in a petri dish and thoroughly compacted to ensure no light leakage. Before capturing hyperspectral images, some preheating steps were performed. The specific steps are as follows: In order to remove baseline drift, the equipment was warmed for thirty minutes before official data was collected. A total of 105 hyperspectral images were collected.

### 2.5 Hyperspectral feature extraction and image feature extraction

#### 2.5.1 ROI acquisition

Through ENVI 5.3 software, spectral extraction was performed on 105 hyperspectral images (Zhang et al., 2023). For each tea sample, 10 regions of interest (ROIs) were randomly selected from the hyperspectral image. Randomly and evenly select each ROI area, ensuring that all areas are rectangular and of the same size, and extract the spectral data from the selected regions. The mean of the 10 sets of data was calculated as the spectral data for the tea sample.

#### 2.5.2 Hyperspectral data correction

A lens cap was placed over the camera lens for the purpose of collecting dark current (B) once it had preheated. Each scan involved moving the lens over a white calibration board to obtain a white calibration (W) image. The hyperspectral data extracted using ENVI software was corrected using the following formula to obtain the final spectral data for modeling, resulting in a total of 105 sets of hyperspectral data.

(2) $$I=\frac{I_{0}-B}{W-B}$$

W is the image of the diffuse reflectance panel, I represent the corrected hyperspectral image, B is the acquired dark-current image, and I_0_ is the original hyperspectral image in the formula. The impact of uneven illumination and dark currents on the hyperspectral image can be effectively reduced by this correction process.

#### 2.5.3 Extraction of color and texture features of tea

The image processing program for extracting the image features of tea samples was developed using the GUI module in MATLAB. We randomly selected 10 ROIs from the hyperspectral image of each tea sample, resulting in 10 sets of image features. Each dataset included nine color features and five texture features. The color attributes comprise the mean values for the following channels: red (R), green (G), blue (B), hue (H), saturation (S), visible light (V), ultra-green transform (2G-R-B)***,*** ratio of red value to green value (R/G), and color angle (hab*).The texture features included mean smoothness (r), entropy (e), average gray value (m), standard deviation (δ), and consistency (U). The mean of the 10 sets of features was taken as the final value, resulting in a total of 105 sets of color and texture feature data.

### 2.6 Multispectral data-fusion strategy

Data fusion reduces the noise and instability of single-source information by integrating different data sources (such as near-infrared spectroscopy and hyperspectral imaging), expanding the spectral range to enhance the model's capability for identification and quantitative analysis (An et al., 2022; Lines et al., 2020). In order to categorize various fresh tea leaf kinds and anticipate chemical indicators, Luo and colleague (Luo, Sun, He, Zhu, & Li, 2023) fused visual and near-infrared data, with good results. In this study, texture feature data (TEX) and spectral data in the 400–1000 nm (VI) and 1000–1580 nm (NI) ranges were fused (NI, VI, NI + VI, NI + TEX, VI + TEX, NI + VI + TEX). In particular, textural feature data and preprocessed spectral information were fused, and the resulting fused data were then processed using a normalization technique to remove the magnitude influence on the modeling outcomes. Table S1 provides an illustration of the abbreviations used in this investigation for different data-fusion techniques.

### 2.7 Data preprocessing

To reduce noise and baseline drift caused by instrumental or external factors, spectral data in the ranges of 400–1000 nm (hyperspectral) and 1000–1580 nm (NIR) were retained for analysis. The retained spectral data is used for model building. Four preprocessing algorithms are employed to reduce issues such as noise and baseline drift in near-infrared and hyperspectral data. The algorithms used include: Savitzky–Golay (S–G) smoothing (Yang et al., 2021), multiplicative scatter correction (MSC) (Sun et al., 2019), one-dimensional median filtering (Medfilt) (Dong et al., 2020), and normalization (z-score) (Dong et al., 2021).

The S-G preprocessing algorithm reduces noise in near-infrared and hyperspectral data while preserving key spectral features, enhancing data quality and analytical accuracy. MSC can remove light scattering effects from spectral data, thereby improving data stability and enhancing the quality of spectral data. The medfilt algorithm replaces each value with the median of its neighboring values to remove noise from the spectral data, helping to preserve edges while smoothing out anomalies. This algorithm effectively reduces random noise and improves the consistency of the data. The z-score algorithm standardizes the data by centering it around zero and scaling it based on the standard deviation, helping to eliminate variations caused by different scales in the spectral data. By using these preprocessing algorithms, noise and baseline drift can be effectively addressed, which is crucial for the performance of subsequent data analysis or machine learning models.

### 2.8 Establish classification and quantitative prediction models

The data used for model development included the near-infrared spectral data, hyperspectral data, and color texture feature data of 105 tea samples mentioned in Sections 2.4.1, 2.5.2, and 2.5.3. Due to the large number of features in the data, we applied Principal Component Analysis (PCA) before building the model. PCA was used to reduce the dimensionality of the data, significantly decreasing the model's input while retaining the primary information. The main operation of PCA involved selecting the optimal number of principal components (PCs) based on the minimum error (RMSEC) from the calibration set, and this number of PCs was used as input for the qualitative classification model. After dimensionality reduction via PCA, the Kennard-Stone (K—S) method (Luo et al., 2021) was used to divide these datasets into 80 training sets and 25 prediction sets.

#### 2.8.1 Establishment of origin discrimination model

In this study, traditional machine learning models, including Random Forest (RF) (Dai et al., 2023), Particle Swarm Optimization-based Support Vector Machine (PSO-SVM) (Li et al., 2024; Yang et al., 2024), and deep learning Convolutional Neural Network (CNN) (Mao et al., 2023), were used to classify the spectra of tea samples from six different regions. Since Support Vector Machine (SVM) can address the nonlinear relationship between class labels and input variables, has fewer hyperparameters, and a simple structure, we chose the Radial Basis Function (RBF) kernel as the kernel function for SVM. This method was developed using the Li-SVMLAB toolbox in the MATLAB environment. Random Forest is an algorithm based on decision trees that improves model accuracy by integrating multiple Classification and Regression Trees (CART models) through an ensemble approach (ntree). The number of decision trees (N) directly affects the accuracy of the Random Forest model. In this study, an optimization process was performed with 40 different values of N (ranging from 50 to 1000, with a step size of 25). Interestingly, the deep learning CNN model used a convolutional kernel size of 2 × 1, ReLU activation function, max-pooling layer size of 2 × 1, stride of [2,1], and a fully connected layer to output the number of classes, flattening the input data into a single dimension.

#### 2.8.2 Establishment of key intrinsic component prediction models

The regression models used in this study include linear partial least squares regression (PLSR) (Liu et al., 2022; Liu, Yang, Luo, Bin, & Dong, 2021), nonlinear support vector regression (SVR), random forest (RF) (Zhang et al., 2024), and convolutional neural network (CNN) models. Linear partial least squares regression (PLSR) is a commonly used multivariate regression method that is effective at handling high-dimensional data. It can extract useful information from multiple influencing factors, resulting in a high level of model robustness and accuracy.

#### 2.8.3 Model evaluation indicators

Using five-fold cross-validation on the training set, we were able to establish the ideal number of principle components for PLSR, the number of decision trees for RF, and the penalty coefficient for SVR—regardless of whether the model was for prediction or classification. The established models were assessed using the following final model performance evaluation indicators: relative percentage difference (RPD), root mean square error of prediction (RMSEP), root mean square error of calibration (RMSEC), Calibration set correlation coefficient(*Rc*)and prediction set correlation coefficient (Rp). It is generally believed that the larger the Rc and Rp values are and the closer they are to 1, and the smaller and closer RMSEC and RMSEP are, the better the model performance is considered. The model's poor predictive ability when RPD < 1.4, its limited ability to generate precise quantitative predictions for the target when RPD is between 1.4 and 1.8, its good predictive performance when RPD is between 1.8 and 2.0, and its strong predictive ability when RPD > 2.0 are particularly noteworthy (He et al., 2023). These indicators help us evaluate the prediction accuracy, fitting ability, and generalization capability of the models from multiple perspectives, ultimately selecting the most suitable model for identifying Rizhao green tea and predicting key intrinsic components.

### 2.9 Data analysis software

Descriptive statistical analysis and analysis of variance were performed using IBM SPSS Statistics 27. Plotting was conducted using Origin 2022. S-G, MSC, medfilt, z-score, PLSR, RF, SVM, SVR, and CNN were all coded and executed in MATLAB 2019a.

## 3 Results and analysis

### 3.1 Sensory evaluation, physicochemical composition, and color texture analysis of Rizhao green tea

**Fig. 1:**
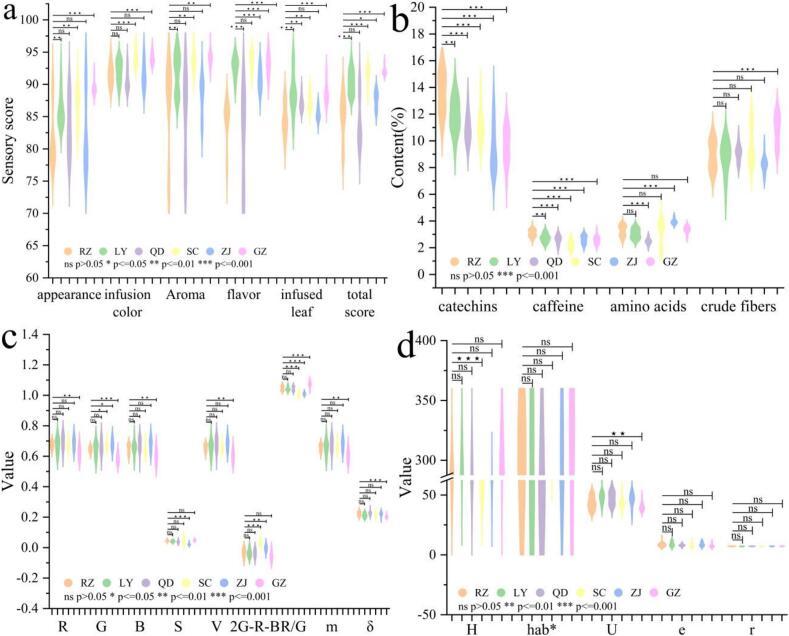
The sensory score, physicochemical composition and color texture characteristics of tea samples from six regions were analyzed by violin plot and correlation test among different regions. (a) Sensory evaluation data; (b) Key physicochemical component content; (c) Color characteristics; (d) Texture characteristics.

The sensory five-factor evaluation data and physicochemical composition of RZ, LY, QD, SC, ZJ, and GZ from different regions were tested using the Shapiro–Wilk test, with each group having a *p*-value greater than 0.05, indicating that each group of data follows a normal distribution. Therefore, single-factor ANOVA variance analysis, Bonferroni, and Tamhane posthoc multiple comparison tests were conducted. Violin plots were created for sensory evaluation and physicochemical composition data of tea samples (Fig. 1), depicting their content and probability density distribution to make the data more intuitive.

#### 3.1.1 Sensory evaluation data analysis

As shown in Fig. 1a, the appearance score of RZ was 81.7 ± 4.5, which was significantly lower than that of LY (86.2 ± 2.7), QD (83.6 ± 4.4), SC (94.2 ± 2.9), ZJ (82.7 ± 6.2), and GZ (89.5 ± 1.0). The average flavor score of RZ (83.8 ± 3.6) was lower than that of LY (92.7 ± 1.6) and ZJ (90.8 ± 1.8) but higher than that of QD (81.9 ± 7.1), SC (93.6 ± 1.4), and GZ (93.1 ± 1.7). RZ exhibited significant (*P* < 0.01) and extremely significant (*P* < 0.001) differences in appearance and flavor compared to LY, SC, and GZ, whereas no significant difference (*P* > 0.05) was observed between RZ and QD. This may be attributed to the proximity of Rizhao and Qingdao and the similarity in tea processing techniques, leading to a similar appearance and flavor with a richer taste than green teas from other provinces. The infusion color score of RZ was 91.5 ± 1.7, falling between LY (92.1 ± 1.7) and SC (94.2 ± 1.3), as well as QD (90.8 ± 1.7) and GZ (84.1 ± 0.9), and was nearly identical to that of ZJ (91.5 ± 2.1). RZ exhibited an extremely significant difference in infusion color compared to SC and GZ (*P* < 0.001). The infusion color of Sichuan and Guizhou green teas was bright and tender green, whereas that of Rizhao green tea was bright yellow-green. The aroma score of RZ was 86.5 ± 7.2, lower than that of LY (91.2 ± 3.1), SC (93.7 ± 1.8), ZJ (88.1 ± 3.0), and GZ (94.2 ± 1.2) but higher than that of QD (85.0 ± 6.9). Rizhao green tea exhibited a significant difference in aroma compared to Sichuan and Guizhou green teas (*P* < 0.01). The infused leaf score of RZ was 84.6 ± 2.5, lower than that of LY (88.9 ± 3.1), SC (87.4 ± 1.3), and GZ (88.8 ± 1.7) but higher than that of QD (87.1 ± 1.2) and ZJ (85.4 ± 1.0). There was no significant difference in infused leaf between Rizhao and Zhejiang green teas (*P* > 0.05), whereas significant (P < 0.01) or extremely significant (*P* < 0.001) differences were observed between Rizhao and the other four green teas. Geographically, Rizhao green tea is grown in a high-latitude tea-producing region, whereas Guizhou, Sichuan, and Zhejiang green teas are produced in southern tea regions. Differences in growth environment, soil nutrients, air temperature, and humidity contribute to the significant or extremely significant differences in sensory flavor between Rizhao green tea and low-latitude southern green teas.

#### 3.1.2 Analysis of physicochemical composition data of Rizhao green tea

As shown in Fig. 1b, RZ exhibits significant (*P* < 0.01) or extremely significant differences (*P* < 0.001) in catechins, caffeine, and amino acid content compared to green teas from the other five regions, while there is no significant difference (*P* > 0.05) in crude fiber content compared to SC and ZJ. Catechins and caffeine influence the strength and richness of tea infusion, with RZ having average catechins and caffeine contents of 13.6 % and 3.1 %, respectively, both higher than those of the other five regions. This indicates that RZ has a “stronger” flavor, consistent with the sensory evaluation conclusion that RZ has a higher richness than southern teas. Amino acids contribute to the fresh taste of tea infusion, with RZ having an average amino acid content of 3.3 %, lower than ZJ (4.0 %) but higher than the other four regions, indicating that RZ has a more pronounced and fresher flavor. The crude fiber content of RZ is 9.1 %, lower than that of GZ (10.9 %). This demonstrates that RZ tea has a rich composition of intrinsic components, which accounts for its strong infusion resistance.

#### 3.1.3 Analysis of color and texture characteristics of Rizhao green tea

As shown in Fig. 1c–d, RZ and GZ exhibit significant (P < 0.01) or extremely significant (P < 0.001) differences in R, G, B, V, R/G, m, δ, and U, while the three green teas from Shandong Province (RZ, LY, and QD) show no significant differences (P > 0.05) in color and texture characteristics. Rizhao tea and Laoshan green tea from Qingdao are both located in northern China and grow in mountainous regions with relatively humid climates and distinct seasons. The similar growing environments may result in similar basic colors and texture characteristics of tea leaves (Sun et al., 2023). Moreover, the two teas share many similarities in processing techniques, leading to similar colors and textures in the final product.

### 3.2 Hierarchical clustering and correlation analysis of sensory and internal quality components of Rizhao green tea

**Fig. 2:**
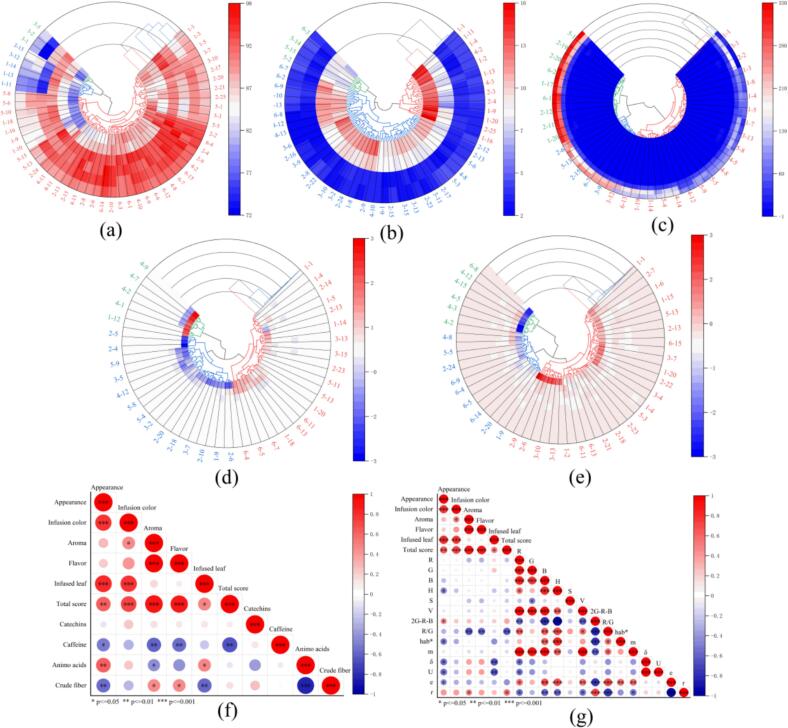
Hierarchical clustering heatmap based on (a) sensory scores, (b) physicochemical components, (c) color texture features, (d) The first ten PCs of the hyperspectrum and (e)The first ten PCs of the near infrared spectrum; (f) Pearson correlation coefficient heat maps of sensory scores and physicochemical components; (g) Pearson correlation coefficient heat maps of sensory scores and color texture features.

First, the Kolmogorov–Smirnov test (K—S test) was conducted for each group of sensory scores and physicochemical composition data. The results demonstrated that the significance level (P) was greater than 0.05, indicating that each group of data followed a normal distribution. The relationship between the sensory evaluations of Rizhao green tea and its physicochemical composition, color, and texture attributes was examined using Pearson's two-tailed test (Fig. 2f–g). The findings showed that while free amino acids showed a significant positive correlation with appearance and infused leaf but a significant negative correlation with aroma, caffeine exhibited a significant negative correlation with appearance, aroma, flavor, and overall sensory scores (*P* < 0.01). Crude fiber showed a strong positive link with flavor and aroma and a substantial negative correlation with appearance and infused leaf. This suggests that the main internal components of Rizhao green tea influence its sensory scores. As can be seen from Fig. 2g, the color feature factor H, texture feature factors hab*, δ, U and e of Rizhao green tea were significantly negatively correlated with its appearance. The color characteristic factor 2G-R-B of Rizhao green tea was positively correlated with its appearance. The color factor R/G was negatively correlated with aroma and taste. The texture characteristic factors δ and U was negatively correlated with infused leaf.

To further explore the differences between Rizhao green tea and other types of tea in terms of sensory, physicochemical, color, and texture features, PCA was conducted on near-infrared and hyperspectral data. Subsequently, HCA was performed on the first 10 principal components (PCs) based on Euclidean distance. Since the contribution rates of the first 10 PCs were 99.91 % and 99.99 %, they represented almost all the sample information. The samples were divided into six classes based on different regions, with each sample labeled (1–1 representing the first sample of Rizhao green tea, 2–1 representing the first sample of Linyi green tea, and so on). Fig. 2a–e illustrates the HCA results of the five sensory evaluation scores, key physicochemical components, color and texture characteristics, the first ten principal components (PCs) of hyperspectral principal component analysis, and the first ten PCs of near-infrared spectral principal component analysis. They exhibited poor clustering results, with different categories misclassified.

### 3.3 Near-infrared and hyperspectral analysis of Rizhao green tea

**Fig. 3:**
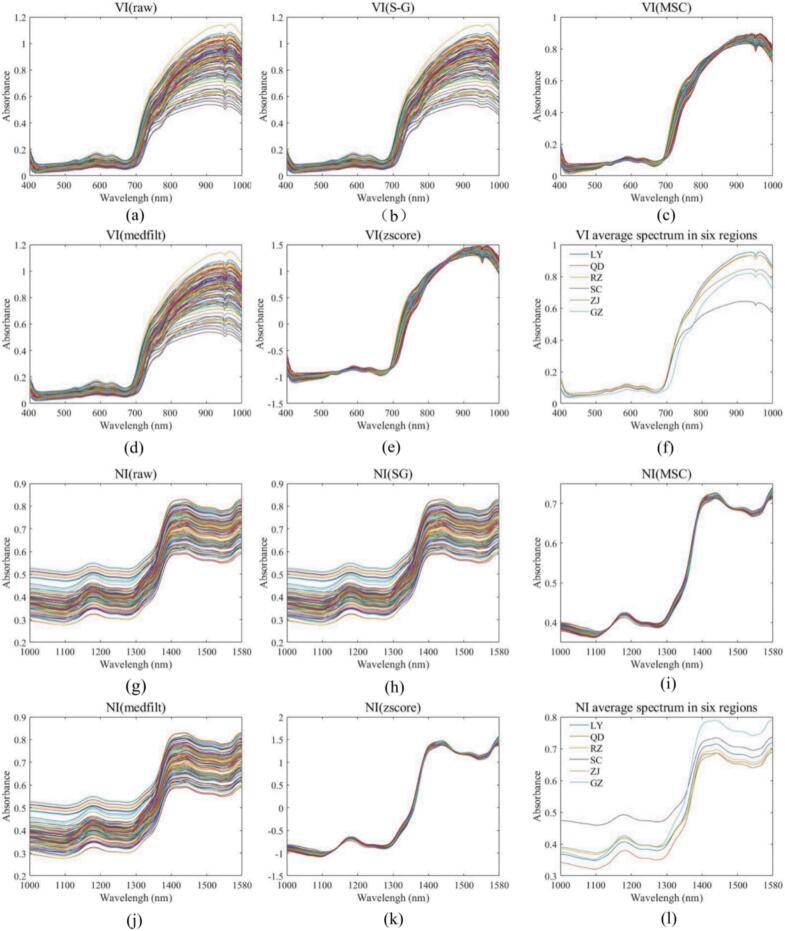
Original and four different pretreatment methods for VI and NI spectra. Original, S-G, MSC, medfilt, and z-score preprocessed spectrograms of VI(a-e); VI average spectrum in six regions(f) Original, S-G, MSC, medfilt, and z-score preprocessed spectrograms of NI(g-k); NI average spectrum in six regions(l).

The average near-infrared (NIR) spectrum of Rizhao green tea is shown in Fig. 3l. Several distinct absorption peaks can be observed in the spectrum, which are primarily attributed to the molecular vibration characteristics of chemical components in the tea leaves. The absorption peak near 1160–1180 nm is associated with the second overtone vibrations of C—H and N—H bonds in the molecules of Rizhao green tea. The C—H bond is related to fatty substances and alkanes (e.g., waxes and lipids) in tea. The waxy layer on the tea leaf surface contains fatty substances whose C—H bonds exhibit weak absorption in this range. The N—H bond is related to proteins and amino acids (e.g., theanine) in tea. The NIR spectral values of Rizhao green tea rise sharply in the 1300–1380 nm range, mainly due to the second overtone absorption of O—H groups. This indicates the presence of a certain amount of bound water in the tea samples and reflects the interaction between polar molecules (such as proteins, tea polyphenols, and carbohydrates) and water in the tea. A strong absorption peak near 1430 nm corresponds to the first overtone absorption of O—H bonds in water molecules, representing the stretching vibration of hydroxyl (O—H) bonds. The 1430–1470 nm range exhibits weak C—H absorption, corresponding to the first overtone stretching vibration of C—H bonds. The 1470–1500 nm range represents the first overtone absorption of N—H bonds, corresponding to the stretching vibration of amino (N—H) groups. Finally, the 1500–1580 nm range is primarily characterized by the first overtone absorption of C—H bonds, associated with the stretching vibrations of carbon‑hydrogen bonds, which may be related to volatile aromatic components in tea (Song, Wenqing Yi, Liu, Wang, & Ning, 2025; Zhang, Yang, Ruan, & Chen, 2024).

The average hyperspectral curve of Rizhao green tea is shown in Fig. 3f. In the visible light range, it primarily reflects the changes of chlorophyll and related pigments. Rizhao green tea exhibits a strong absorption peak at 520 nm, which is mainly attributed to chlorophyll *b* and carotenoids, reflecting the tenderness and antioxidant capacity of the tea leaves. The absorption peak around 580 nm is associated with tea polyphenols and their oxidation products, reflecting the degree of oxidation and the formation of aromatic compounds in the tea leaves. The strong absorption peak near 630 nm is related to chlorophyll derivatives formed by the photothermal degradation of chlorophyll *a*, corresponding to the conjugated C=H double bond. Beyond 700 nm, water molecules in the tea leaves exhibit a characteristic absorption of near-infrared light, which significantly affects the spectral values, reaching a peak near 900 nm. The absorption peak around 940 nm is caused by the overtone or combination modes of the bending vibration of the O—H bond in water molecules. The hydrogen bonds between water molecules alter the position and intensity of the O—H absorption band, leading to changes in the width and shape of the absorption peak near 940 nm (Li et al., 2024).

### 3.4 Identification model of Rizhao green tea

A comparative evaluation of origin discrimination models was conducted using Support Vector Machine (SVM), Random Forest (RF), and Convolutional Neural Network (CNN) models. Table 1 presents the evaluation metrics for selecting the optimal origin discrimination model for Rizhao green tea. The confusion matrices for the training and test sets of the SVM (Fig. S1), RF (Fig. S2), and CNN (Fig. S3) classification models were plotted. A notable number of misclassifications were concentrated among green teas from Rizhao, Qingdao, and Linyi within Shandong Province. Across all classification models, non-Shandong green teas were misclassified only three times: once in the training set of the SVM model based on VI (Fig. S1(a)) and twice in the test sets of the CNN models based on VI + TEX and VI + NI + TEX (Fig. S3(*h*,*l*)). The difficulty in distinguishing green teas from different regions within Shandong in some models is likely due to their similar growing environments, minimal spectral differences, and comparable color and texture characteristics. As shown in Table 1, the training and test accuracy of the SVM model based on NI, the RF models based on NI and VI + NI + TEX, and the CNN models based on NI and VI + NI all reached 100 %. Among the SVM, RF, and CNN models, those based on NI data achieved higher accuracy than those based on VI. However, NI-based data demonstrated superior discriminative ability for tea variety and origin. This suggests that the near-infrared (NI) spectral range is more relevant to the vibrational and rotational energy level transitions of tea's internal molecules and carries rich structural and compositional information. Therefore, near-infrared spectroscopy is a more suitable choice for tea origin discrimination. However, models incorporating VI + TEX and NI + TEX exhibited slightly lower accuracy, indicating that adding color and texture features did not enhance the ability to distinguish the origin of Rizhao green tea. Considering that CNN algorithms involve complex computations and require additional computational resources, the most optimal origin discrimination models for Rizhao green tea are the SVM model based on NI data and the RF classification model based on NI and VI + NI + TEX.

**Table 1:** The performance (accuracy of classification) of different data fusion methods in the three classification models.

Model	Data fusion methods	PCs	Calibration set		Prediction set	
			Result	CCR	Result	CCR
SVM	VI	18	69/80	86.25 %	20/25	80 %
	NI	13	80/80	100 %	25/25	100 %
	VI + NI	23	80/80	100 %	24/25	96 %
	VI + TEX	27	80/80	100 %	22/25	88 %
	NI + TEX	15	80/80	100 %	24/25	96 %
	VI + NI + TEX	23	80/80	100 %	22/25	88 %

RF	VI	18	80/80	100 %	24/25	96 %
	NI	13	80/80	100 %	25/25	100 %
	VI + NI	17	80/80	100 %	24/25	96 %
	VI + TEX	27	80/80	100 %	23/25	92 %
	NI + TEX	15	80/80	100 %	22/25	88 %
	VI + NI + TEX	23	80/80	100 %	25/25	100 %

CNN	VI	18	80/80	100 %	24/25	96 %
	NI	13	80/80	100 %	25/25	100 %
	VI + NI	17	80/80	100 %	25/25	100 %
	VI + TEX	27	80/80	100 %	21/25	84 %
	NI + TEX	15	80/80	100 %	22/25	88 %
	VI + NI + TEX	23	80/80	100 %	23/25	92 %

SVM: Support vector machine; RF: random forest; CNN: Convolutional Neural Networks; CCR: correct classification rate.

### 3.5 Optimal preprocessing for prediction models

To minimize noise, improve model accuracy, and remove unnecessary information from the raw spectra, four preprocessing techniques were applied. The original near-infrared spectra, hyperspectral images, and the average spectra of six origins after different preprocessing methods were plotted (Fig. 3). Catechins, caffeine, amino acids, crude fiber, and the total sensory score were used as prediction outputs. Various data fusion methods were preprocessed, and the fused data underwent PCA for dimensionality reduction. The minimum number of principal components (PCs) was selected as the input dimensionality for the model. A support vector machine (SVM) prediction model was established to predict key intrinsic components of Rizhao green tea (catechins, caffeine, amino acids, and crude fiber) as well as the total sensory score, with model evaluation metrics presented in Table S2–Table S6. Severe overfitting was observed in most models, making it difficult to determine an optimal preprocessing algorithm due to the complexity of combining four preprocessing methods with six data fusion approaches. Therefore, the optimal preprocessing and data fusion combination was selected based on the model evaluation metrics. By comparing model evaluation metrics, the best preprocessing and data fusion method for each physicochemical index and total sensory score was highlighted in bold. The final selections were as follows: VI + MSC was the optimal combination for predicting catechin content, with an RPD of 2.010. VI + NI + medfilt was the best combination for predicting caffeine content, with an RPD of 1.761. NI + TEX was the optimal combination for predicting amino acid content, with an RPD of 2.800. VI + NI + medfilt was the best combination for predicting crude fiber content, with an RPD of 2.815. NI + medfilt was the optimal combination for predicting the total sensory score, with an RPD of 4.277.

### 3.6 Model selection for each quality indicator

**Fig. 4:**
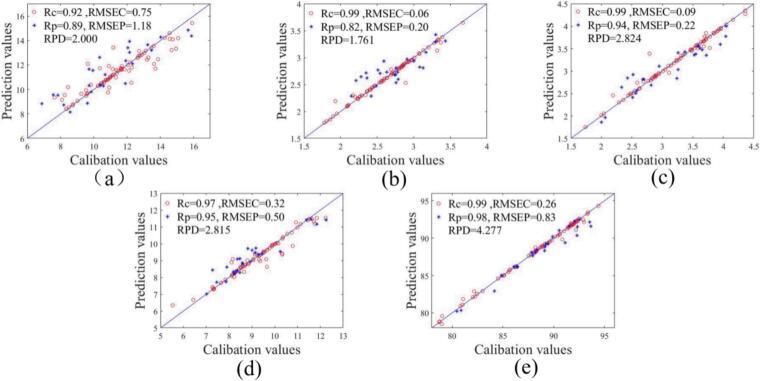
Scatter plot of the model prediction value and actual value of catechins model (a), caffeine model (b), animo acids model (c), crude fiber model (d) and sensory score model (e).

Due to the influence of various factors, including both linear and nonlinear effects, during the acquisition of near-infrared and hyperspectral data, an optimal modeling approach is needed for the rapid detection of sensory scores and physicochemical indicators of Rizhao green tea. Based on the optimal combinations selected in the previous section, predictive models were developed using linear partial least squares regression (PLSR), nonlinear random forest (RF), and convolutional neural network (CNN) methods, with further optimization applied to refine the prediction models. Table 2 displays the outcomes. It can be observed that among all prediction models, the SVR nonlinear model demonstrated the highest level of performance. Due to the presence of a large number of high-dimensional features in near-infrared and hyperspectral data, which exhibit complex nonlinear relationships with the target variables, the prediction accuracy of nonlinear models are consistently higher than that of linear model. SVR can capture complex nonlinear relationships in high-dimensional data through kernel functions. Additionally, SVR minimizes generalization error by identifying the optimal margin of tolerance, making it more robust when dealing with small sample sizes. This explains why the SVR model established in this study outperforms the RF model. Finally, we determined that the best prediction model for catechin content was VI + MSC + SVR, as depicted in Fig. 4a, where Rc was 0.92, Rp was 0.89, and PRD was 2.000. The best prediction model for caffeine content was VI + NI + medfilt+SVR, as depicted in Fig. 4b, where Rc was 0.99, Rp was 0.82, and PRD was 1.761. The best prediction model for amino acid content was NI + TEX+SVR, as depicted in Fig. 4c, where Rc was 0.99, Rp was 0.94, and PRD was 2.824. The best prediction model for crude fiber content was VI + NI + medfilt+SVR, as depicted in Fig. 4d, where Rc was 0.97, Rp was 0.95, and PRD was 2.815. The best prediction model for total sensory scores was NI + medfilt+SVR, as depicted in Fig. 4e, where Rc was 0.99, Rp was 0.98, and PRD was 4.277.

**Table 2:** Prediction results of different PLSR, SVR, RF and CNN models for quality indicators.

Paraments	Data fusion	Data preprocessing	Methods	PCs	Calibration set		Prediction set		RPD
					Rc	RMSEC	Rp	RMSEP	
Catechins	VI	MSC	PLSR	5	0.42	1.70	0.71	1.97	0.435
			SVR	5	0.92	0.75	0.89	1.18	2.000
			RF	5	0.95	0.79	0.84	1.58	1.513
			CNN	30	0.99	0.25	0.48	1.40	1.391
Caffeine	VI + NI	medfilt	PLSR	14	0.41	0.39	0.68	0.27	0.897
			SVR	14	0.99	0.06	0.82	0.20	1.761
			RF	14	0.98	0.16	0.77	0.26	1.592
			CNN	30	0.99	0.08	0.69	0.21	1.782
Animo acids	NI + TEX	zscore	PLSR	18	0.73	0.42	0.91	0.26	2.057
			SVR	18	0.99	0.09	0.94	0.22	2.824
			RF	18	0.99	0.23	0.91	0.44	1.667
			CNN	30	0.99	0.09	0.40	0.38	1.287
Crude fiber	VI + NI	medfilt	PLSR	12	0.70	0.93	0.83	0.86	1.216
			SVR	12	0.97	0.32	0.95	0.50	2.815
			RF	12	0.98	0.49	0.92	0.85	1.720
			CNN	30	0.99	0.35	0.45	0.79	1.350
Sensory score	NI	medfilt	PLSR	7	0.66	3.01	0.72	2.76	1.213
			SVR	7	0.99	0.26	0.98	0.83	4.277
			RF	7	0.98	1.06	0.95	1.43	2.898
			CNN	30	0.99	0.78	0.73	2.34	1.943

## 4 Conclusions

This study used multispectral combined with color and texture features of tea samples in a total of six data-fusion methods, to achieve the traceability of Rizhao green tea origin. We conducted a one-way ANOVA variance analysis on the sensory evaluation and physicochemical data of tea samples from six different regions across China. Rizhao green tea exhibited significant or extremely significant differences in sensory flavor compared to tea from low-latitude regions in the south. The content of catechins, caffeine, and amino acids in Rizhao green tea differed significantly or extremely significantly from green tea in the other five regions, with overall higher levels of physicochemical components compared to the other five regions.

Three different classification algorithms were successfully used to distinguish Rizhao green tea from teas in other geographical regions of China. The optimal classification models, NI + SVM，NI + RF and VI + NI + TEX, were selected, both achieving an accuracy of 100 %. We established SVR models for total sensory scores and physicochemical components (catechins, caffeine, amino acids, and crude fiber) using six different data-fusion methods and four different preprocessing methods, selecting the best combinations. Finally, we compared the linear PLSR, nonlinear RF, and CNN prediction models using the selected best combinations and identified the best models. The results indicated that VI + MSC + SVR was the best prediction model combination for catechins, VI + NI + medfilt+SVR for caffeine, NI + TEX+z-score + SVR for amino acids, VI + NI + medfilt+SVR for crude fiber, and NI + medfilt+SVR for total sensory scores. Their relative percent deviation (RPD) values were 2.000, 1.761, 2.824, 2.815, and 4.277, respectively, indicating that the models had excellent predictive performance. This study demonstrates that the combination of VNHI and machine learning deep learning algorithms with chemometrics for the origin traceability of Rizhao green tea is highly effective.

## CRediT authorship contribution statement

**Mengqi Guo:** Writing – original draft, Data curation. **Zhiwei Chen:** Software, Methodology, Conceptualization. **Zezhong Ding:** Investigation, Formal analysis. **Dewen Wang:** Validation, Investigation. **Dandan Qi:** Visualization. **Min Lu:** Resources, Project administration. **Mei Wang:** Writing – review & editing. **Chunwang Dong:** Supervision, Resources, Methodology, Funding acquisition, Conceptualization.

## Ethical statement

Sensory evaluation is an analytical method based on human perception to assess tea quality. This study did not involve medical interventions, behavioral manipulation, or genetic research on humans or animals, nor did it pose any risks to the evaluators' safety, privacy, or health; thus, ethical approval was not required. No human ethics committee or formal documentation process was available for this work but the authors confirm that the appropriate protocols for protecting the rights and privacy of all participants were utilized during the execution of the research, with no coercion to participate, full disclosure of study requirements and risks. Participants provided verbal informed consent prior to taking part in the study. There will be no release of participant data without their knowledge, and they have the ability to withdraw from the study at any time.

## Declaration of competing interest

No conflicts of interest exist in the submission of this manuscript, and the manuscript is approved by all authors for publication. All the authors listed have approved the manuscript that is enclosed.

## Data Availability

Data will be made available on request.

## Appendix A Supplementary data

Supplementary material Image 1
