# Supplementary material for: Traceability of Rizhao green tea origin based on multispectral data fusion strategy and chemometrics
Source: Food Chem X. 2025 Mar 18;27:102346. doi: 10.1016/j.fochx.2025.102346 (PMC11982953; doi:10.1016/j.fochx.2025.102346)
Supplement: Supplementary file 1 — Supplementary material [file mmc1.docx]

**Supplementary Material**

**Fig. S1.** Confusion matrix of training set and prediction set based on SVM model for 6 kinds of data fusion. In a-b, the input is VI; In c-d, the input is NI; In e-f, the input is VI+NI; In g-h, the input is VI+TEX; In i-j, the input is NI+TEX; In k-l, the input is VI+NI+TEX.

**Fig. S2.** Confusion matrix of training set and prediction set based on RF model for 6 kinds of data fusion. In a-b, the input is VI; In c-d, the input is NI; In e-f, the input is VI+NI; In g-h, the input is VI+TEX; In i-j, the input is NI+TEX; In k-l, the input is VI+NI+TEX.

**Fig. S3.** Confusion matrix of training set and prediction set based on CNN model for 6 kinds of data fusion. In a-b, the input is VI; In c-d, the input is NI; In e-f, the input is VI+NI; In g-h, the input is VI+TEX; In i-j, the input is NI+TEX; In k-l, the input is VI+NI+TEX.

**Table S1.** Abbreviations of different data fusion methods and corresponding instructions.

**Table S2.** Effects of different preprocessing methods on Catechins prediction model SVR.

**Table S3.** Effects of different preprocessing methods on Caffeine prediction model SVR.

**Table S4.** Effects of different preprocessing methods on Animo acids prediction model SVR.

**Table S5.** Effects of different preprocessing methods on Crude fiber prediction model SVR.

**Table S6.** Effects of different preprocessing methods on Sensory score prediction model SVR.


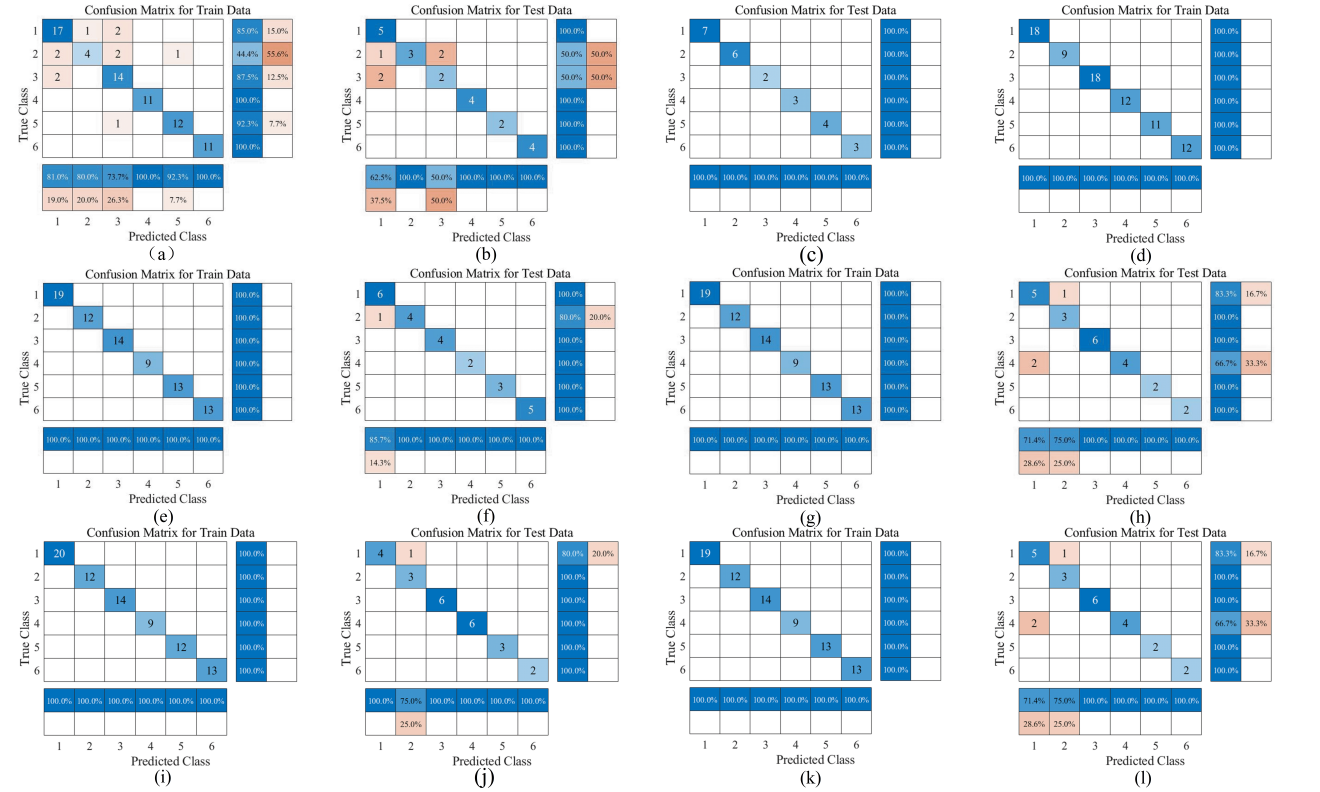


Fig. S1. Confusion matrix of training set and prediction set based on SVM model for 6 kinds of data fusion. In a-b, the input is VI; In c-d, the input is NI; In e-f, the input is VI+NI; In g-h, the input is VI+TEX; In i-j, the input is NI+TEX; In k-l, the input is VI+NI+TEX.


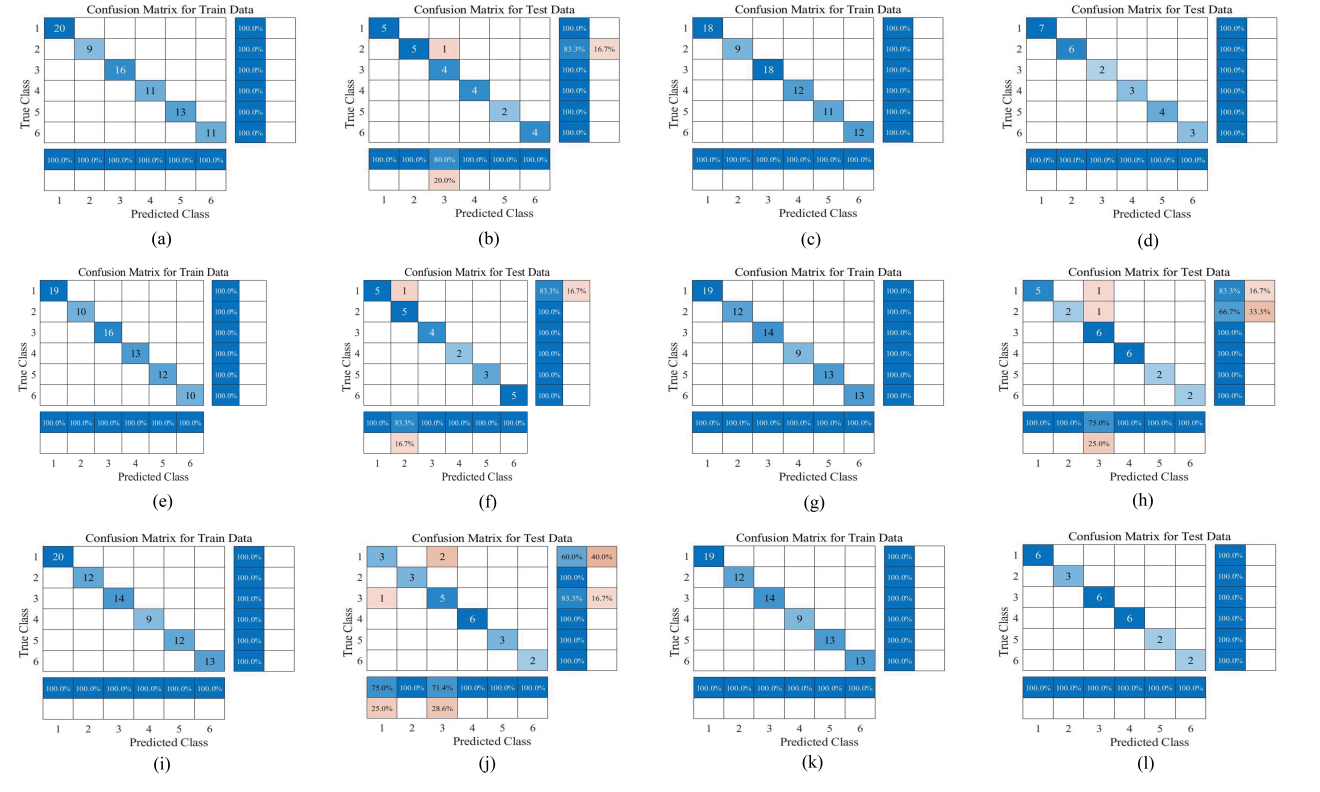


Fig. S2. Confusion matrix of training set and prediction set based on RF model for 6 kinds of data fusion. In a-b, the input is VI; In c-d, the input is NI; In e-f, the input is VI+NI; In g-h, the input is VI+TEX; In i-j, the input is NI+TEX; In k-l, the input is VI+NI+TEX.


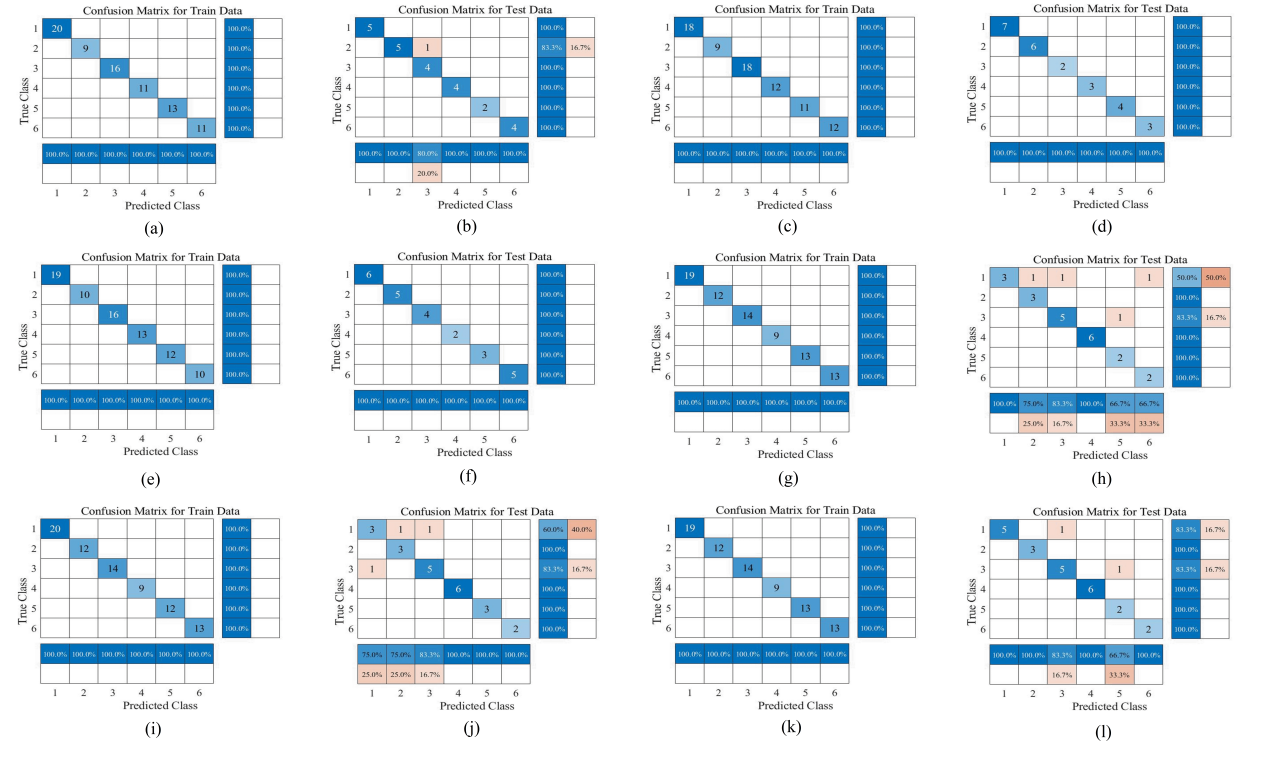


Fig. S3. Confusion matrix of training set and prediction set based on CNN model for 6 kinds of data fusion. In a-b, the input is VI; In c-d, the input is NI; In e-f, the input is VI+NI; In g-h, the input is VI+TEX; In i-j, the input is NI+TEX; In k-l, the input is VI+NI+TEX.

Table S1. Abbreviations of different data fusion methods and corresponding instructions.

| Abbreviation | instructions |
| --- | --- |
| VI | Spectral data from 400 to 1000 nm |
| NI | Spectral data from 1000 to 1580 nm |
| VI+NI | The fusion data of VI and NI |
| VI+TEX | The fusion data of VI and TEX |
| NI+TEX | The fusion data of NI and TEX |
| VI+NI+TEX | The fusion data of VI, NI and TEX |

Table S2. Effects of different preprocessing methods on Catechins prediction model SVR.

| Paraments | Data fusion | Data preprocessing | PCs | Calibration set | | Prediction set | | RPD |
| --- | --- | --- | --- | --- | --- | --- | --- | --- |
|  |  |  |  | Rc | RMSEC | Rp | RMSEP |  |
| Catechins | VI | Raw | 5 | 0.99 | 0.13 | 0.74 | 1.69 | 1.255 |
|  |  | S-G | 13 | 0.99 | 0.17 | 0.75 | 1.47 | 1.263 |
|  |  | **MSC** | **5** | **0.92** | **0.75** | **0.89** | **1.18** | **2.010** |
|  |  | Medfilt | 4 | 0.63 | 1.49 | 0.62 | 1.82 | 1.256 |
|  |  | Zscore | 6 | 0.84 | 1.03 | 0.87 | 1.55 | 1.653 |
|  | NI | **Raw** | **11** | **0.99** | **0.06** | **0.67** | **1.50** | **1.357** |
|  |  | S-G | 13 | 0.94 | 0.69 | 0.66 | 1.55 | 1.307 |
|  |  | MSC | 11 | 0.91 | 0.86 | 0.63 | 1.73 | 1.265 |
|  |  | Medfilt | 10 | 0.84 | 1.09 | 0.70 | 1.52 | 1.346 |
|  |  | Zscore | 11 | 0.86 | 1.02 | 0.67 | 1.47 | 1.268 |
|  | VI+NI | Raw | 10 | 0.91 | 0.81 | 0.79 | 1.41 | 1.640 |
|  |  | **S-G** | **16** | **0.97** | **0.46** | **0.81** | **1.31** | **1.670** |
|  |  | MSC | 10 | 0.92 | 0.76 | 0.72 | 1.64 | 1.438 |
|  |  | Medfilt | 6 | 0.92 | 0.80 | 0.72 | 1.41 | 1.366 |
|  |  | Zscore | 10 | 0.91 | 0.82 | 0.74 | 1.53 | 1.454 |
|  | VI+TEX | Raw | 20 | 0.99 | 0.05 | 0.58 | 1.61 | 1.193 |
|  |  | **S-G** | **20** | **0.99** | **0.05** | **0.66** | **1.65** | **1.400** |
|  |  | MSC | 17 | 0.99 | 0.05 | 0.66 | 1.58 | 1.333 |
|  |  | Medfilt | 8 | 0.60 | 1.56 | 0.54 | 1.9 | 1.122 |
|  |  | Zscore | 16 | 0.99 | 0.05 | 0.75 | 1.5 | 1.295 |
|  | NI+TEX | Raw | 13 | 0.76 | 1.32 | 0.63 | 1.56 | 1.192 |
|  |  | S-G | 18 | 0.99 | 0.10 | 0.75 | 1.33 | 1.423 |
|  |  | MSC | 12 | 0.76 | 1.33 | 0.47 | 1.88 | 1.124 |
|  |  | **Medfilt** | **16** | **0.94** | **0.82** | **0.78** | **1.46** | **1.522** |
|  |  | Zscore | 10 | 0.87 | 1.04 | 0.46 | 1.85 | 1.000 |
|  | VI+NI+TEX | Raw | 18 | 0.99 | 0.04 | 0.63 | 1.56 | 1.230 |
|  |  | S-G | 23 | 0.97 | 0.50 | 0.71 | 1.57 | 1.416 |
|  |  | MSC | 13 | 0.99 | 0.11 | 0.47 | 2.21 | 1.000 |
|  |  | Medfilt | 10 | 0.60 | 1.54 | 0.49 | 2.00 | 1.093 |
|  |  | **Zscore** | **17** | **0.97** | **0.51** | **0.75** | **1.60** | **1.426** |

Table S3. Effects of different preprocessing methods on Caffeine prediction model SVR.

| Paraments | Data fusion | Data preprocessing | PCs | Calibration set | | Prediction set | | RPD |
| --- | --- | --- | --- | --- | --- | --- | --- | --- |
|  |  |  |  | Rc | RMSEC | Rp | RMSEP |  |
| Caffeine | VI | Raw | 6 | 0.87 | 0.22 | 0.55 | 0.30 | 1.190 |
|  |  | S-G | 13 | 0.99 | 0.03 | 0.59 | 0.27 | 1.127 |
|  |  | **MSC** | **6** | **0.99** | **0.05** | **0.73** | **0.34** | **1.452** |
|  |  | Medfilt | 8 | 0.99 | 0.01 | 0.72 | 0.26 | 1.350 |
|  |  | Zscore | 5 | 0.94 | 0.13 | 0.66 | 0.33 | 1.300 |
|  | NI | Raw | 10 | 0.94 | 0.16 | 0.63 | 0.26 | 1.283 |
|  |  | S-G | 12 | 0.95 | 0.14 | 0.52 | 0.30 | 1.086 |
|  |  | MSC | 9 | 0.93 | 0.16 | 0.71 | 0.25 | 1.278 |
|  |  | Medfilt | 7 | 0.88 | 0.20 | 0.72 | 0.25 | 1.305 |
|  |  | **Zscore** | **11** | **0.92** | **0.18** | **0.68** | **0.23** | **1.364** |
|  | VI+NI | Raw | 11 | 0.99 | 0.06 | 0.80 | 0.24 | 1.683 |
|  |  | S-G | 15 | 0.99 | 0.01 | 0.78 | 0.25 | 1.585 |
|  |  | MSC | 9 | 0.98 | 0.09 | 0.78 | 0.25 | 1.560 |
|  |  | **Medfilt** | **14** | **0.99** | **0.06** | **0.82** | **0.20** | **1.761** |
|  |  | Zscore | 11 | 0.98 | 0.07 | 0.81 | 0.26 | 1.732 |
|  | VI+TEX | Raw | 13 | 0.99 | 0.02 | 0.63 | 0.34 | 1.307 |
|  |  | S-G | 22 | 0.99 | 0.01 | 0.58 | 0.31 | 1.240 |
|  |  | MSC | 9 | 0.73 | 0.29 | 0.25 | 0.34 | 1.006 |
|  |  | **Medfilt** | **11** | **0.98** | **0.07** | **0.75** | **0.32** | **1.365** |
|  |  | Zscore | 5 | 0.96 | 0.12 | 0.50 | 0.36 | 1.066 |
|  | NI+TEX | Raw | 16 | 0.93 | 0.15 | 0.70 | 0.30 | 1.401 |
|  |  | S-G | 22 | 0.99 | 0.05 | 0.73 | 0.27 | 1.303 |
|  |  | MSC | 12 | 0.81 | 0.24 | 0.47 | 0.31 | 1.127 |
|  |  | **Medfilt** | **11** | **0.99** | **0.02** | **0.76** | **0.31** | **1.458** |
|  |  | Zscore | 15 | 0.99 | 0.02 | 0.77 | 0.28 | 1.400 |
|  | VI+NI+TEX | Raw | 15 | 0.99 | 0.02 | 0.74 | 0.28 | 1.501 |
|  |  | S-G | 19 | 0.96 | 0.13 | 0.68 | 0.27 | 1.390 |
|  |  | MSC | 11 | 0.81 | 0.25 | 0.43 | 0.31 | 1.100 |
|  |  | Medfilt | 17 | 0.99 | 0.02 | 0.73 | 0.31 | 1.415 |
|  |  | **Zscore** | **11** | **0.99** | **0.01** | **0.82** | **0.23** | **1.752** |

Table S4. Effects of different preprocessing methods on Animo acids prediction model SVR.

| Paraments | Data fusion | Data preprocessing | PCs | Calibration set | | Prediction set | | RPD |
| --- | --- | --- | --- | --- | --- | --- | --- | --- |
|  |  |  |  | Rc | RMSEC | Rp | RMSEP |  |
| Animo acids | VI | Raw | 15 | 0.99 | 0.01 | 0.89 | 0.33 | 2.091 |
|  |  | **S-G** | **20** | **0.99** | **0.01** | **0.92** | **0.28** | **2.501** |
|  |  | MSC | 12 | 0.99 | 0.03 | 0.86 | 0.31 | 1.895 |
|  |  | Medfilt | 13 | 0.99 | 0.09 | 0.83 | 0.37 | 1.727 |
|  |  | Zscore | 15 | 0.99 | 0.05 | 0.89 | 0.30 | 2.079 |
|  | NI | **Raw** | **13** | **0.98** | **0.13** | **0.91** | **0.22** | **2.466** |
|  |  | S-G | 13 | 0.98 | 0.13 | 0.91 | 0.22 | 2.408 |
|  |  | MSC | 10 | 0.97 | 0.15 | 0.82 | 0.27 | 1.763 |
|  |  | Medfilt | 8 | 0.99 | 0.06 | 0.92 | 0.27 | 2.297 |
|  |  | Zscore | 13 | 0.99 | 0.10 | 0.91 | 0.23 | 2.271 |
|  | VI+NI | Raw | 25 | 0.98 | 0.11 | 0.86 | 0.28 | 2.000 |
|  |  | S-G | 22 | 0.99 | 0.10 | 0.88 | 0.27 | 2.077 |
|  |  | MSC | 28 | 0.95 | 0.18 | 0.87 | 0.35 | 2.040 |
|  |  | **Medfilt** | **13** | **0.99** | **0.03** | **0.92** | **0.24** | **2.561** |
|  |  | Zscore | 21 | 0.96 | 0.16 | 0.85 | 0.33 | 1.904 |
|  | VI+TEX | Raw | 19 | 0.98 | 0.11 | 0.80 | 0.36 | 1.667 |
|  |  | **S-G** | **24** | **0.99** | **0.01** | **0.90** | **0.30** | **2.309** |
|  |  | MSC | 15 | 0.98 | 0.11 | 0.57 | 0.44 | 1.100 |
|  |  | Medfilt | 16 | 0.99 | 0.01 | 0.74 | 0.43 | 1.511 |
|  |  | Zscore | 21 | 0.99 | 0.02 | 0.56 | 0.39 | 1.149 |
|  | NI+TEX | Raw | 20 | 0.97 | 0.14 | 0.89 | 0.27 | 2.197 |
|  |  | S-G | 17 | 0.98 | 0.12 | 0.92 | 0.26 | 2.534 |
|  |  | MSC | 19 | 0.99 | 0.02 | 0.68 | 0.36 | 1.350 |
|  |  | Medfilt | 15 | 0.95 | 0.17 | 0.91 | 0.28 | 2.475 |
|  |  | **Zscore** | **18** | **0.99** | **0.09** | **0.94** | **0.22** | **2.800** |
|  | VI+NI+TEX | **Raw** | **21** | **0.99** | **0.04** | **0.92** | **0.23** | **2.642** |
|  |  | S-G | 28 | 0.99 | 0.09 | 0.94 | 0.31 | 2.293 |
|  |  | MSC | 11 | 0.94 | 0.22 | 0.59 | 0.39 | 1.200 |
|  |  | Medfilt | 21 | 0.99 | 0.01 | 0.86 | 0.32 | 2.000 |
|  |  | Zscore | 16 | 0.99 | 0.09 | 0.92 | 0.22 | 2.562 |

Table S5. Effects of different preprocessing methods on Crude fiber prediction model SVR.

| Paraments | Data fusion | Data preprocessing | PCs | Calibration set | | Prediction set | | RPD |
| --- | --- | --- | --- | --- | --- | --- | --- | --- |
|  |  |  |  | Rc | RMSEC | Rp | RMSEP |  |
| Crude fiber | VI | Raw | 9 | 0.98 | 0.28 | 0.74 | 0.81 | 1.492 |
|  |  | S-G | 19 | 0.99 | 0.06 | 0.80 | 0.72 | 1.710 |
|  |  | MSC | 7 | 0.98 | 0.28 | 0.85 | 0.80 | 1.852 |
|  |  | **Medfilt** | **16** | **0.99** | **0.21** | **0.91** | **0.57** | **2.190** |
|  |  | Zscore | 8 | 0.99 | 0.23 | 0.82 | 0.66 | 1.780 |
|  | NI | Raw | 10 | 0.99 | 0.08 | 0.88 | 0.57 | 2.103 |
|  |  | S-G | 10 | 0.99 | 0.08 | 0.88 | 0.57 | 2.102 |
|  |  | **MSC** | **9** | **0.96** | **0.38** | **0.91** | **0.58** | **2.231** |
|  |  | Medfilt | 11 | 0.99 | 0.19 | 0.87 | 0.56 | 1.970 |
|  |  | Zscore | 10 | 0.98 | 0.28 | 0.86 | 0.61 | 1.957 |
|  | VI+NI | Raw | 10 | 0.99 | 0.10 | 0.72 | 0.96 | 1.451 |
|  |  | S-G | 23 | 0.95 | 0.41 | 0.80 | 0.81 | 1.716 |
|  |  | MSC | 11 | 0.96 | 0.39 | 0.85 | 0.73 | 1.852 |
|  |  | **Medfilt** | **12** | **0.97** | **0.32** | **0.95** | **0.50** | **2.815** |
|  |  | Zscore | 10 | 0.97 | 0.31 | 0.83 | 0.72 | 1.709 |
|  | VI+TEX | Raw | 14 | 0.98 | 0.27 | 0.77 | 0.90 | 1.557 |
|  |  | S-G | 11 | 0.96 | 0.40 | 0.74 | 0.83 | 1.429 |
|  |  | MSC | 13 | 0.93 | 0.51 | 0.79 | 0.83 | 1.447 |
|  |  | Medfilt | 11 | 0.99 | 0.14 | 0.79 | 0.86 | 1.613 |
|  |  | **Zscore** | **14** | **0.87** | **0.70** | **0.81** | **0.66** | **1.629** |
|  | NI+TEX | Raw | 18 | 0.98 | 0.26 | 0.86 | 0.72 | 1.876 |
|  |  | **S-G** | **19** | **0.97** | **0.33** | **0.91** | **0.64** | **2.086** |
|  |  | MSC | 11 | 0.82 | 0.78 | 0.62 | 0.98 | 1.233 |
|  |  | Medfilt | 14 | 0.99 | 0.13 | 0.87 | 0.70 | 1.840 |
|  |  | Zscore | 19 | 0.99 | 0.12 | 0.83 | 0.72 | 1.800 |
|  | VI+NI+TEX | **Raw** | **16** | **0.94** | **0.47** | **0.92** | **0.65** | **2.138** |
|  |  | S-G | 22 | 0.97 | 0.37 | 0.79 | 0.73 | 1.621 |
|  |  | MSC | 15 | 0.94 | 0.46 | 0.90 | 0.76 | 1.600 |
|  |  | Medfilt | 15 | 0.93 | 0.47 | 0.87 | 0.70 | 2.000 |
|  |  | Zscore | 11 | 0.98 | 0.30 | 0.89 | 0.64 | 2.095 |

Table S6. Effects of different preprocessing methods on Sensory score prediction model SVR.

| Paraments | Data fusion | Data preprocessing | PCs | Calibration set | | Prediction set | | RPD |
| --- | --- | --- | --- | --- | --- | --- | --- | --- |
|  |  |  |  | Rc | RMSEC | Rp | RMSEP |  |
| Sensory score | VI | Raw | 15 | 0.99 | 0.28 | 0.87 | 1.80 | 1.946 |
|  |  | S-G | 15 | 0.99 | 0.30 | 0.91 | 1.55 | 2.260 |
|  |  | MSC | 6 | 0.99 | 0.13 | 0.92 | 1.44 | 2.590 |
|  |  | **Medfilt** | **9** | **0.99** | **0.49** | **0.96** | **1.18** | **3.579** |
|  |  | Zscore | 14 | 0.99 | 0.08 | 0.94 | 1.31 | 2.939 |
|  | NI | Raw | 11 | 0.99 | 0.08 | 0.96 | 1.02 | 3.299 |
|  |  | S-G | 13 | 0.99 | 0.10 | 0.94 | 1.10 | 3.042 |
|  |  | MSC | 9 | 0.99 | 0.23 | 0.97 | 1.07 | 3.904 |
|  |  | **Medfilt** | **7** | **0.99** | **0.26** | **0.98** | **0.83** | **4.277** |
|  |  | Zscore | 13 | 0.99 | 0.14 | 0.90 | 1.54 | 2.257 |
|  | VI+NI | Raw | 11 | 0.99 | 0.39 | 0.95 | 1.20 | 3.373 |
|  |  | S-G | 13 | 0.99 | 0.13 | 0.96 | 1.14 | 3.541 |
|  |  | MSC | 9 | 0.99 | 0.47 | 0.87 | 2.01 | 1.954 |
|  |  | **Medfilt** | **23** | **0.99** | **0.21** | **0.97** | **1.06** | **3.966** |
|  |  | Zscore | 10 | 0.99 | 0.21 | 0.95 | 1.19 | 3.158 |
|  | VI+TEX | **Raw** | **25** | **0.99** | **0.09** | **0.93** | **1.54** | **2.727** |
|  |  | S-G | 21 | 0.99 | 0.32 | 0.85 | 2.10 | 1.811 |
|  |  | MSC | 11 | 0.93 | 1.43 | 0.69 | 3.26 | 1.276 |
|  |  | Medfilt | 12 | 0.99 | 0.09 | 0.87 | 2.12 | 2.067 |
|  |  | Zscore | 7 | 0.90 | 1.58 | 0.57 | 3.83 | 1.155 |
|  | NI+TEX | **Raw** | **20** | **0.99** | **0.67** | **0.93** | **1.51** | **2.778** |
|  |  | S-G | 21 | 0.99 | 0.34 | 0.91 | 1.66 | 2.403 |
|  |  | MSC | 16 | 0.97 | 0.97 | 0.88 | 2.10 | 1.982 |
|  |  | Medfilt | 26 | 0.99 | 0.08 | 0.91 | 1.95 | 2.250 |
|  |  | Zscore | 21 | 0.99 | 0.08 | 0.92 | 1.62 | 2.566 |
|  | VI+NI+TEX | Raw | 13 | 0.88 | 1.90 | 0.82 | 2.24 | 1.761 |
|  |  | S-G | 27 | 0.99 | 0.25 | 0.88 | 1.81 | 1.983 |
|  |  | MSC | 13 | 0.98 | 0.76 | 0.71 | 2.99 | 1.391 |
|  |  | **Medfilt** | **21** | **0.99** | **0.20** | **0.92** | **1.76** | **2.482** |
|  |  | Zscore | 7 | 0.99 | 0.36 | 0.72 | 2.64 | 1.428 |
